# Supplementary material for: Evaluation of a savings-led family-based economic empowerment intervention for AIDS-affected adolescents in Uganda: A four-year follow-up on efficacy and cost-effectiveness
Source: PLoS One. 2019 Dec 31;14(12):e0226809. doi: 10.1371/journal.pone.0226809 (PMC6938344; doi:10.1371/journal.pone.0226809)
Supplement: S1 Appendix — Fig. A. CONSORT Flow Diagram: The Bridges to the Future Study (2011–2017) Table A. Descriptive statistics of adolescent characteristics at baseline Table B. Descriptive statistics on characteristics of attrited sample Table C. Description of outcome measures Table D. Itemized total per-participant costs based on intent-to-treat sample (all costs are in 2012 Ugandan Shillings unless otherwise indicated). (DOCX) [file pone.0226809.s001.docx]

**S1 Appendix**


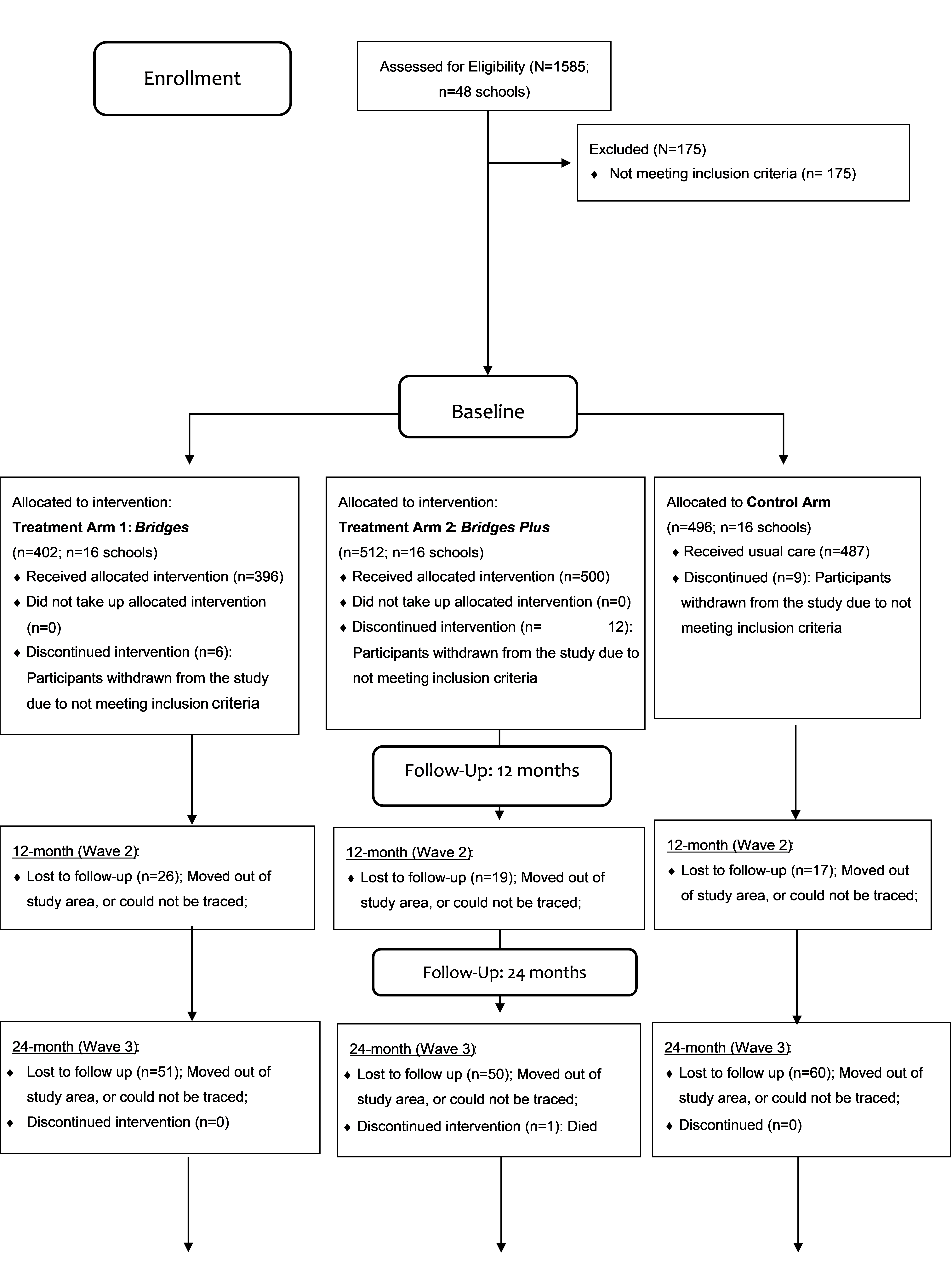


Fig. A. CONSORT Flow Diagram: *The Bridges to the Future Study* (2011-2017)


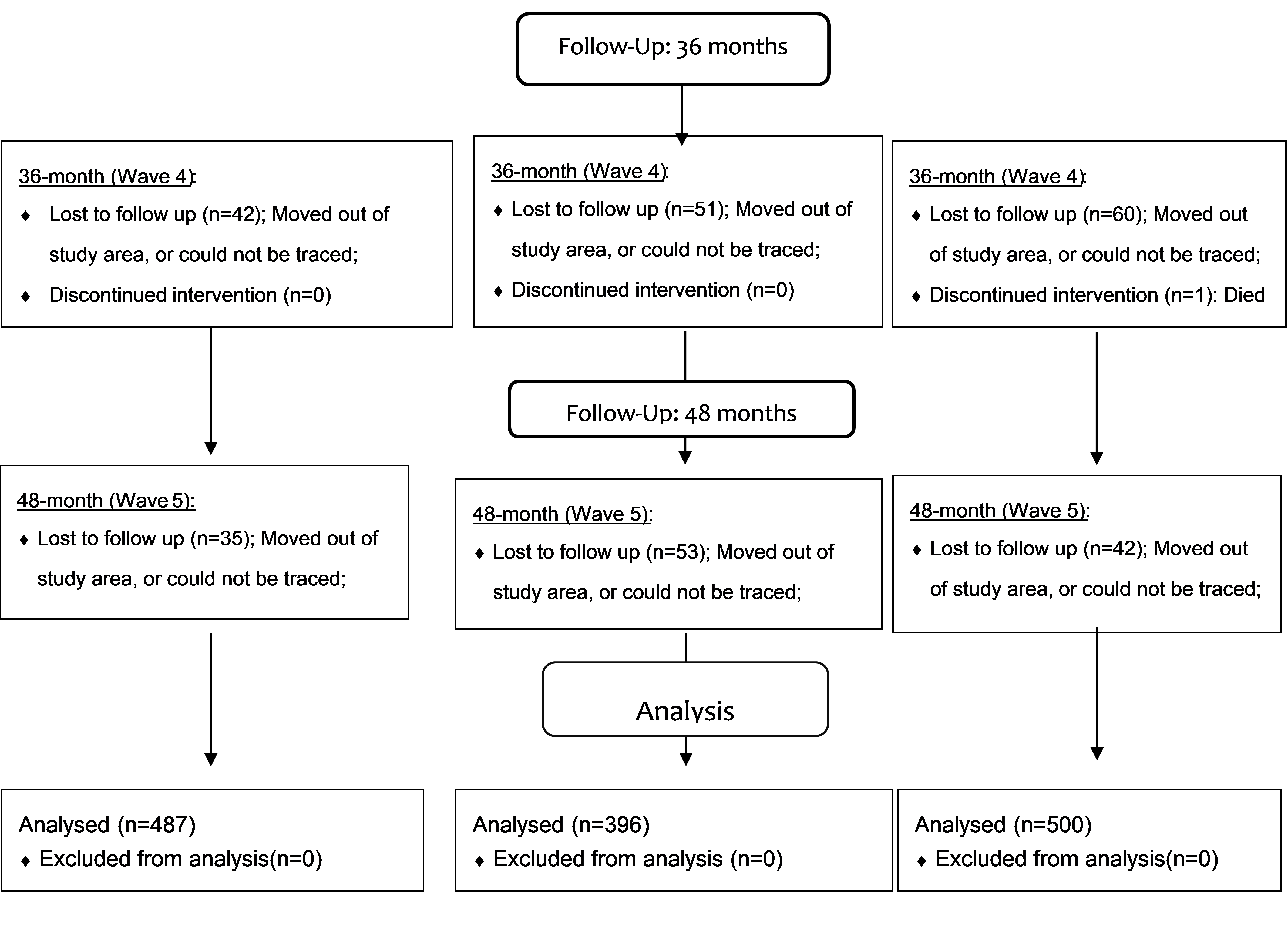


Fig. A. *(continued)* CONSORT Flow Diagram: *The Bridges to the Future Study* (2011-2017)

Table A. Descriptive statistics of adolescent characteristics at baseline

| **Baseline characteristics** | **Usual care** | **Bridges** | **Bridges PLUS** |
| --- | --- | --- | --- |
| **Sociodemographics** |  |  |  |
| Age | 12.75 (1.23) | 12.56 (1.31) | 12.71 (1.25) |
| Female | 55% | 57% | 56% |
| Household size | 6.43 (2.97) | 6.29 (2.62) | 6.32 (2.74) |
| Number of children in household | 3.18 (2.32) | 3.14 (2.08) | 3.23 (2.18) |
| Double orphan | 25% | 18% | 20% |
| Years since living in the household | 7.12 (4.41) | 7.19 (4.44) | 7.44 (4.54) |
| Primary caregiver |  |  |  |
| Parents | 37% | 41% | 44% |
| Grandparents | 40% | 35% | 36% |
| Other relatives | 23% | 24% | 21% |
| **Outcomes** |  |  |  |
| Self-rated health (ranges 1-5) 1 item | 4.28 (.90) | 4.11 (1.00) | 4.13 (.97) |
| Child depression (ranges 0-54) 27 items; α=.68 | 9.72 (5.13) | 9.67 (4.97) | 9.98 (4.97) |
| Hopelessness (range 0-20) 20 items; α=.65 | 5.42 (3.17) | 5.13 (3.20) | 5.51 (2.95) |
| Self-concept (range 20-100) 20 items; α=.74 | 67.49 (9.31) | 67.54 (9.65) | 65.60 (9.15) |
| Self-efficacy (range: 29-116) 29 items; α=.82 | 99.31 (11.17) | 98.22 (12.22) | 96.67 (12.58) |
| Sexual risk-taking intentions (range 5-25) 5 items; α=.76 | 9.02 (5.00) | 9.06 (4.76) | 9.02 (4.60) |
| HIV prevention attitudes (range 5-25) 5 items; α=.70 | 17.54 (6.74) | 18.08 (6.35) | 17.56 (6.53) |
| HIV knowledge (range: 0-16) 16 items; α=.83 | 7.75 (2.85) | 7.91 (2.81) | 7.66 (2.82) |
| **Sample sizes** |  |  |  |
| Baseline | 487 | 396 | 500 |
| 48-months follow-up | 444 | 361 | 444 |

Note: Percentages or means are provided for each indicator, and standard deviations are provided for continuous outcomes in parenthesis.

Table B. Descriptive statistics on characteristics of attrited sample

|  | Retained respondent (n=1249) | | Attritted respondents (n=134) | | Difference |
| --- | --- | --- | --- | --- | --- |
|  | Mean/ % | SD | Mean/ % | SD |  |
| Group status |  |  |  |  |  |
| Control | 35.55 |  | 32.09 |  |  |
| Bridges | 28.90 |  | 26.12 |  |  |
| Bridges PLUS | 35.55 |  | 41.79 |  |  |
| Age | 12.68 | 1.28 | 12.75 | 1.08 |  |
| Female | 53.96 |  | 72.39 |  | *** |
| Household size | 6.35 | 2.74 | 6.40 | 3.18 |  |
| Number of children | 3.18 | 2.15 | 3.27 | 2.63 |  |
| Years living in the household | 7.35 | 4.48 | 6.37 | 4.18 | ** |
| Double orphan | 19.14 |  | 23.13 |  |  |
| Primary caregiver |  |  |  |  |  |
| Parents | 40.43 |  | 26.87 |  | ** |
| Grandparents | 35.39 |  | 50.75 |  | *** |
| Other relatives | 24.18 |  | 22.39 |  |  |

Note: To examine whether children who remained in the survey and those who attritted are different in terms of their demographic and outcome characteristics, we employed a multilevel model for each characteristic and used attrition status to predict that characteristic. In this multilevel model, we included school-level random intercepts to account for clustering at the school level. We report the p-value from the attrition status coefficient in each model/for each characteristic

*p<.05, **p<.01, ***p<.001

Table C. Description of outcome measures

| **Outcomes** | **Description** | **Response options** |
| --- | --- | --- |
| Self-rated health^1^ (Ranges 1-5) 1 item | “At present time, would you say that your  physical health is” | Excellent (5) to very poor (1) |
| Child depression^2^ (Range: 0-54) 27 items; 𝛼=.68 | “I am sad once in a while/many times/all the time;” I do most things okay/I do many things wrong/I do everything wrong;” | 0 being the least depressed; 2 being the most depressed |
| Hopelessness^3^ (Range: 0-20) 20 items; 𝛼=.65 | “I don’t expect to get what I really want;” “I might as well give up because there is nothing I can do about making things better for myself;” “My future seems dark.” | True/False |
| Self-concept^4^ (Range: 20-100) 20 items; 𝛼=.74 | “I like the way I look;” “I’m happy with who I am;” “I feel good most of the time.” | Always true (5) to always false (1) |
| Self-efficacy^5^ (Range: 32-116) 29 items; 𝛼=.82 | “Some kids feel they can understand math if they work at it BUT other kids feel that no matter how hard they work at it, it is still very hard to learn math;” “Some kids feel that they can NOT figure out the answers in school even when they try, BUT other kids feel that they can usually figure out the answers in school if they try;” “Some kids find that even when they try, it is hard to get people their age to like them, BUT other kids think that if they try, they can get people their age to like them. | Pick one statement that fits better and score very true or sort of true (range: 1 to 4, with 4 indicating the highest self-efficacy) |
| Sexual risk-taking intentions^6^ (Range: 5-25) 5 items; 𝛼=.76 | “I believe it’s OK for people my age to have sex with someone they’ve just met;” “I agree that it’s OK to force a girlfriend/boyfriend to have sex even when they don’t want to;” “I believe it’s OK to have sex without protection with someone you know.” | Always (5) to never (1) |
| HIV prevention attitudes^7^ (Range: 5-25) 5 items; 𝛼=.70 | “I think it is very important to use condoms every time one has sex;” “As a teenager I think AIDS is a threat to my health;” “I think all people my age who have sex should use condoms.” | Agree a great deal (5) to not at all agree (1) |
| HIV knowledge^8^ (range: 0-16) 16 items; α=.83 | “Sharing needles or syringes (empiso) with an HIV infected person;” “A pregnant woman who has HIV/AIDS can give her unborn baby the virus;”” Not having sexual intercourse with anyone.” | Safe /true (3) or unsafe (2) or not sure (1) |

Table D. Itemized total per-participant costs based on intent-to-treat sample (all costs are in 2012 Ugandan Shillings unless otherwise indicated)

|  | | **Usual Care** | **Bridges** | **BridgesPLUS** |
| --- | --- | --- | --- | --- |
| **Recurrent costs** | | | | |
| **Food aid** | | 28,064 | 28,064 | 28,064 |
| **Educational materials** | | 61,900 | 61,900 | 61,900 |
| **Counseling** | | 39 | 39 | 39 |
| **Recruitment of participants** | | 5,847 | 5,847 | 5,847 |
| **Child savings account** | |  |  |  |
|  | Account opening | - | 23,664 | 23,664 |
|  | Initial account deposit | - | 20,000 | 20,000 |
|  | Annual matched savings | - | 20,776 | 43,105 |
| **Mentorship** | | - | 35,721 | 35,721 |
| **Financial education and income generating activity training** | | - | 37,284 | 37,284 |
| **Personnel (staff salary and allowances)** | | 104,244 | 208,488 | 208,488 |
| **Volunteer and donated resources** | | 3,535 | 404,462 | 404,462 |
| **Capital costs** | | | | |
| **Furniture, equipment and vehicles** | | 67,404 | 134,808 | 134,808 |
| **Total intent-to-treat (ITT) per-participant costs** | | **271,034** | **981,054** | **1,003,383** |
| **Total ITT per-participant costs (in 2012 USD)** | | **103** | **363** | **372** |

References

1. Frankenberg, E., & Jones, N. R. (2004). Self-rated health and mortality: does the relationship extend to a low income setting?. *Journal of health and social behavior*, *45*(4), 441-452.
2. Saylor, C. F., Finch, A. J., Spirito, A., & Bennett, B. (1984). The Children's Depression Inventory: A systematic evaluation of psychometric properties. *Journal of consulting and clinical psychology*, *52*(6), 955.
3. Beck, A. T., Weissman, A., Lester, D., & Trexler, L. (1974). The measurement of pessimism: the hopelessness scale. *Journal of consulting and clinical psychology*, *42*(6), 861.
4. Fitts, W. H., & Roid, G. H. (1988). Tennessee Self-Concept Scale (TSCS): Revised manual. *Los Angeles, CA: Western Psychological Services*.
5. Earls, F., & Visher, C. A. (1997). *Project on human development in Chicago neighborhoods: a research update*. Washington, DC: US Department of Justice, Office of Justice Programs, National Institute of Justice.

Ssewamala FM, Ismayilova L, McKay M, et al. Gender and the effects of an economic empowerment program on attitudes toward sexual risk-taking among AIDS-orphaned adolescent Youth in Uganda. J Adolesc Health 2010;46:372e8

Ssewamala FM, Ismayilova L. Integrating children’s savings accounts in the care and support of orphaned adolescents in rural Uganda. Soc Serv Rev 2009;83:453e72. <https://doi.org/10.1086/605941>.

Ssewamala FM, Alicea S, Bannon W, Ismayilova L. A novel economic intervention to reduce HIV risks among school-going AIDS-orphaned children in rural Uganda. J Adolesc Health 2008;42:102e4
