# Supplementary material for: Evaluation of a savings-led family-based economic empowerment intervention for AIDS-affected adolescents in Uganda: A four-year follow-up on efficacy and cost-effectiveness
Source: PLoS One. 2019 Dec 31;14(12):e0226809. doi: 10.1371/journal.pone.0226809 (PMC6938344; doi:10.1371/journal.pone.0226809)
Supplement: Attachment — Submitted filename: PLOS_Supplementary Material_07.24.2019_GF.docx [file pone.0226809.s003.docx]

**Supplementary material**

Figure S1. CONSORT Flow Diagram: *The Bridges to the Future Study* (2011-2017)

Figure S1. *(continued)* CONSORT Flow Diagram: *The Bridges to the Future Study* (2011-2017)

Table S1. Descriptive statistics of adolescent characteristics at baseline

| **Baseline characteristics** | **Usual care** | **Bridges** | **Bridges PLUS** |
| --- | --- | --- | --- |
| **Sociodemographics** |  |  |  |
| Age | 12.75 (1.23) | 12.56 (1.31) | 12.71 (1.25) |
| Female | 55% | 57% | 56% |
| Household size | 6.43 (2.97) | 6.29 (2.62) | 6.32 (2.74) |
| Number of children in household | 3.18 (2.32) | 3.14 (2.08) | 3.23 (2.18) |
| Double orphan | 25% | 18% | 20% |
| Years since living in the household | 7.12 (4.41) | 7.19 (4.44) | 7.44 (4.54) |
| Primary caregiver |  |  |  |
| Parents | 37% | 41% | 44% |
| Grandparents | 40% | 35% | 36% |
| Other relatives | 23% | 24% | 21% |
| **Outcomes** |  |  |  |
| Self-rated health (ranges 1-5) 1 item | 4.28 (.90) | 4.11 (1.00) | 4.13 (.97) |
| Child depression (ranges 0-54) 27 items; α=.68 | 9.72 (5.13) | 9.67 (4.97) | 9.98 (4.97) |
| Hopelessness (range 0-20) 20 items; α=.65 | 5.42 (3.17) | 5.13 (3.20) | 5.51 (2.95) |
| Self-concept (range 20-100) 20 items; α=.74 | 67.49 (9.31) | 67.54 (9.65) | 65.60 (9.15) |
| Self-efficacy (range: 29-116) 29 items; α=.82 | 99.31 (11.17) | 98.22 (12.22) | 96.67 (12.58) |
| Sexual risk-taking intentions (range 5-25) 5 items; α=.76 | 9.02 (5.00) | 9.06 (4.76) | 9.02 (4.60) |
| HIV prevention attitudes (range 5-25) 5 items; α=.70 | 17.54 (6.74) | 18.08 (6.35) | 17.56 (6.53) |
| HIV knowledge (range: 0-16) 16 items; α=.83 | 7.75 (2.85) | 7.91 (2.81) | 7.66 (2.82) |
| **Sample sizes** |  |  |  |
| Baseline | 487 | 396 | 500 |
| 48-months follow-up | 444 | 361 | 444 |

Note: Percentages or means are provided for each indicator, and standard deviations are provided for continuous outcomes in parenthesis.

Table S2. Description of outcome measures

| **Outcomes** | **Description** | **Response options** |
| --- | --- | --- |
| Self-rated health (Ranges 1-5) 1 item | “At present time, would you say that your  physical health is” | Excellent (5) to very poor (1) |
| Child depression (Range: 0-54) 27 items; 𝛼=.68 | “I am sad once in a while/many times/all the time;” I do most things okay/I do many things wrong/I do everything wrong;” | 0 being the least depressed; 2 being the most depressed |
| Hopelessness (Range: 0-20) 20 items; 𝛼=.65 | “I don’t expect to get what I really want;” “I might as well give up because there is nothing I can do about making things better for myself;” “My future seems dark.” | True/False |
| Self-concept (Range: 20-100) 20 items; 𝛼=.74 | “I like the way I look;” “I’m happy with who I am;” “I feel good most of the time.” | Always true (5) to always false (1) |
| Self-efficacy (Range: 32-116) 29 items; 𝛼=.82 | “Some kids feel they can understand math if they work at it BUT other kids feel that no matter how hard they work at it, it is still very hard to learn math;” “Some kids feel that they can NOT figure out the answers in school even when they try, BUT other kids feel that they can usually figure out the answers in school if they try;” “Some kids find that even when they try, it is hard to get people their age to like them, BUT other kids think that if they try, they can get people their age to like them. | Pick one statement that fits better and score very true or sort of true (range: 1 to 4, with 4 indicating the highest self-efficacy) |
| Sexual risk-taking intentions (Range: 5-25) 5 items; 𝛼=.76 | “I believe it’s OK for people my age to have sex with someone they’ve just met;” “I agree that it’s OK to force a girlfriend/boyfriend to have sex even when they don’t want to;” “I believe it’s OK to have sex without protection with someone you know.” | Always (5) to never (1) |
| HIV prevention attitudes (Range: 5-25) 5 items; 𝛼=.70 | “I think it is very important to use condoms every time one has sex;” “As a teenager I think AIDS is a threat to my health;” “I think all people my age who have sex should use condoms.” | Agree a great deal (5) to not at all agree (1) |
| HIV knowledge (range: 0-16) 16 items; α=.83 | “Sharing needles or syringes (empiso) with an HIV infected person;” “A pregnant woman who has HIV/AIDS can give her unborn baby the virus;”” Not having sexual intercourse with anyone.” | Safe /true (3) or unsafe (2) or not sure (1) |
